# Supplementary material for: Dynamic changes of lung sRAGE in mice with chronic obstructive pulmonary disease induced by cigarette smoke exposure
Source: PLoS One. 2024 Nov 26;19(11):e0313872. doi: 10.1371/journal.pone.0313872 (PMC11594483; doi:10.1371/journal.pone.0313872)
Supplement: Attachment — Submitted filename: Reviewer Attachment.docx [file pone.0313872.s002.docx]

1. **Is the manuscript technically sound, and do the data support the conclusion?**

Yes

- Histopathological, inflammatory markers used to identify the cigarette smoke impact and its sRAGE correlation is technically sound and the conclusion is in line with their findings.

2. **Has the statistical analysis been performed appropriately and rigorously?**

- Yes
- Strength
  - Much of the statistical analysis are appropriate and rigorous, ANOVA and LSD test helped to differentiate which groups are specifically different
- To be clarified or corrected
  - Has all the data fulfilled the ANOVA assumptions especially the normal distribution, if so it would be better to be mentioned, the same to the Pearson analysis.

3. **Have the authors made all data underlying the findings in their manuscript fully available?**

- Yes

4. **Is the manuscript presented in an intelligible fashion and written in standard English?**

Yes

Minor corrections –

- Abstract TNF-αin should be TNF-α in
- Clinical Research(320.6750.19092-6) should be Clinical Research (320.6750.19092-6)
- You used different line spaces at “These results showed that cigarette smoke exposure could induce obstructive ventilatory dysfunction characterized by emphysema in mice, and lung function decreased with prolonged…”
- 15th day should be 15^th^
- Use Pearson correlation … should be Pearson correlation analysis was used …
- Tobacco smoking as an most important etiologic… should be as the most
- Some of the discussion paragraphs are too long and cumbersome, it would be better if fragmented in a certain way and made concise as much as possible

5. **Review comments to the Author**

- The paper is rigorously made and touched areas that needs further exploration and found to be a harbinger around the use of sRAGE as a pharmacological target. It worth’s to find a way to reach the wider scientific community.
