# Supplementary material for: Dynamic changes of lung sRAGE in mice with chronic obstructive pulmonary disease induced by cigarette smoke exposure
Source: PLoS One. 2024 Nov 26;19(11):e0313872. doi: 10.1371/journal.pone.0313872 (PMC11594483; doi:10.1371/journal.pone.0313872)
Supplement: Attachment — Submitted filename: Response to Reviewers.docx [file pone.0313872.s003.docx]

Dear Editors and Reviewers:

Thanks for your letter and the comments suggestions of reviewers concerning our manuscript entitled “Dynamic changes of lung sRAGE in mice with chronic obstructive pulmonary disease induced by cigarette smoke exposure” (PONE-D-24-20210). All these comments are valuable and very helpful for the revision and improvement of our paper. We have studied comments carefully and have made amendments which we hope will be approval.

In addition, we are sorry to tell you that we made a mistake in our manuscript. Six mice in each group were written in the previous manuscript. In fact, there were three mice in each group, and all statistics were carried out according to the three mice, and the data statistical analysis was guaranteed to be effective. All review comments and questions have been corrected in this submission, and the documentation of all original data has been submitted.

**Responding to editors**

1.

**Editors' Comments to Author:**

Please ensure that your manuscript meets PLOS ONE's style requirements, including those for file naming.

**Response to comments:**

We have carefully re-formatted the manuscript and changed the naming of the figures to suit PLOS ONE's style requirements. Ensure that it conforms to the style of your publication.

2.

**Editors' Comments to Author:**

To comply with PLOS ONE submissions requirements, in your Methods section, please provide additional information regarding the experiments involving animals and ensure you have included details on (1) methods of sacrifice, (2) methods of anesthesia and/or analgesia, and (3) efforts to alleviate suffering.

**Response to comments:**

We have amended the methods section of the manuscript for animal experiments to ensure that it includes detailed information on the methods of sacrifice, the methods of anesthesia and the efforts made to alleviate suffering.

3.

**Editors' Comments to Author:**

Please remove any funding-related text from the manuscript and let us know how you would like to update your Funding Statement.

**Response to comments:**

We have removed any funding-related text from the manuscript. The funding statement is accurate and no changes need to be made.

4.

**Editors' Comments to Author:**

Please confirm at this time whether or not your submission contains all raw data required to replicate the results of your study. Authors must share the “minimal data set” for their submission.

**Response to comments:**

We've submitted all raw data required to replicate the results of our study and upload them as a Supporting Information file.

5.

**Editors' Comments to Author:**

When completing the data availability statement of the submission form, you indicated that you will make your data available on acceptance. We strongly recommend all authors decide on a data sharing plan before acceptance, as the process can be lengthy and hold up publication timelines. Please note that, though access restrictions are acceptable now, your entire data will need to be made freely accessible if your manuscript is accepted for publication. This policy applies to all data except where public deposition would breach compliance with the protocol approved by your research ethics board. If you are unable to adhere to our open data policy, please kindly revise your statement to explain your reasoning and we will seek the editor's input on an exemption. Please be assured that, once you have provided your new statement, the assessment of your exemption will not hold up the peer review process.

**Response to comments:**

We have modified our statement and decide to join the data sharing plan before acceptance.

6.

**Editors' Comments to Author:**

PLOS requires an ORCID iD for the corresponding author in Editorial Manager on papers submitted after December 6th, 2016. Please ensure that you have an ORCID iD and that it is validated in Editorial Manager.

**Response to comments:**

Thank you for pointing this out, the corresponding author has provided the ORCID iD and it has been validated in Editorial Manager.

7.

**Editors' Comments to Author:**

Please review your reference list to ensure that it is complete and correct. If you have cited papers that have been retracted, please include the rationale for doing so in the manuscript text, or remove these references and replace them with relevant current references. Any changes to the reference list should be mentioned in the rebuttal letter that accompanies your revised manuscript. If you need to cite a retracted article, indicate the article’s retracted status in the References list and also include a citation and full reference for the retraction notice.

**Response to comments:**

We have checked all references in the manuscript to ensure that they are complete and accurate. For the references section, we made a modification by changing "曹君, 陈平, 杨悦, 欧阳若芸, 彭红. 烟雾暴露所致肺气肿小鼠模型的建立与评价. 中国实验动物学报. 2010;18: 278-282+366" to "Cao J, Chen P, Yang Y, Ouyang RY, Peng H. Establishment and Assessment of a Mouse Model of Cigarette Smoke-Induced Emphysema. Acta Laboratorium Animalis Scientia Sinica. 2010;18: 278-282+366".

**Responding to reviewers**

1.

**Reviewers ' Comments to Author:**

It may make it more clear in the Methods section 1.3.1 Establishment of mouse model in the second sentence to state that 6 mice were sacrificed after each time point instead of only wring n=6 at the end of the sentence.

**Response to comments:**

We removed the statement here and recharacterized our grouping in clearer terms.

2.

**Reviewers ' Comments to Author:**

In the abstract, indicate what the acronym CS stands for so it is clear to your audience.

**Response to comments:**

In the abstract of our manuscript, we indicated what the acronym CS stands for.

3.

**Reviewers ' Comments to Author:**

In the Discussion in the 11th line (7th sentence) RAGE is misspelled as RGAE.

**Response to comments:**

Thank you for your careful review, we have corrected the typos.

4.

**Reviewers ' Comments to Author:**

Abstract TNF-αin should be TNF-α in.

**Response to comments:**

We have fixed the error here.

5.

**Reviewers ' Comments to Author:**

Clinical Research(320.6750.19092-6) should be Clinical Research (320.6750.19092-6).

**Response to comments:**

We have fixed the error here.

6.

**Reviewers ' Comments to Author:**

You used different line spaces at “These results showed that cigarette smoke exposure could induce obstructive ventilatory dysfunction characterized by emphysema in mice, and lung function decreased with prolonged…”.

**Response to comments:**

We have corrected the formatting error here.

7.

**Reviewers ' Comments to Author:**

15th day should be 15^th^.

**Response to comments:**

We have fixed the error here.

8.

**Reviewers ' Comments to Author:**

Use Pearson correlation … should be Pearson correlation analysis was used …

**Response to comments:**

We have fixed the error here.

9.

**Reviewers ' Comments to Author:**

Tobacco smoking as an most important etiologic… should be as the most.

**Response to comments:**

We have fixed the error here.

10.

**Reviewers ' Comments to Author:**

Some of the discussion paragraphs are too long and cumbersome, it would be better if fragmented in a certain way and made concise as much as possible.

**Response to comments:**

We have streamlined some of the discussion sections and added new subsections to make them easier to read.

We deeply appreciate your consideration of our manuscript, and we look forward to receiving your follow-up comments!

Sincerely,

Qiang WANG
